# An atypical case of hemodialysis access stent migration 

**DOI:** 10.5414/CNCS110737

**Published:** 2022-01-24

**Authors:** Juan C. Duque, Marwan Tabbara, Laisel Martinez, Karen Manzur-Pineda, Roberto I. Vazquez-Padron, Adriana Dejman

**Affiliations:** 1 Katz Family Division of Nephrology and Hypertension, and; 2 DeWitt Daughtry Family Department of Surgery, Division of Vascular and Endovascular Surgery, University of Miami Miller School of Medicine, Miami, FL, USA

**Keywords:** stent fracture, stent migration, hemodialysis vascular access

## Abstract

Endovascular stent fractures are commonly seen in arteries but are rare events in the venous system. Stents deployed in hemodialysis vascular accesses can fracture and migrate to proximal locations. Complications associated with stent fracture include in-stent stenosis and central vein stenosis. In this report, we present a unique case of a hemodialysis access stent fracture that migrated to the left ventricle and manifested with chest pain.

## Background 

Cardiovascular mortality is significantly higher in chronic kidney disease (CKD) and end-stage kidney disease (ESKD) patients than in the general population [1], especially in patients who experience a cardiovascular event [1]. There is an increased rate of myocardial infarction, heart failure, arrhythmias, and death in this population [2, 3]. Frequent causes of acute chest pain in ESKD patients include myocardial infarction, pericarditis, pleuritis, air embolism, gastroesophageal reflux, and complications from dialysis catheters [4], but infrequently a complication from a functional arteriovenous fistula (AVF) or graft (AVG). A functional AVF is by far the preferred vascular conduit for hemodialysis [5]. 

Hemodialysis vascular accesses require, in most cases, multiple interventions to keep them functional, and the last line of intervention for AVG is the placement of endoluminal stents [6]. Percutaneous transluminal angioplasty (PTA) is the gold standard treatment for stenotic lesions in AVG [7]. The use of stents is frequently seen with good clinical results [8], but there is a potential risk of harming the access and losing the vasculature available for a secondary fistula creation in the same extremity [6]. There are three well described indications for stent placement in a dialysis vascular access: a recoil lesion, a rupture, and recurrent stenosis. There are three types of stents available for endovascular intervention including bare metal, stent-graft, and drug-coated stent [9]. From these, bare metal stents are generally avoided [7] but sometimes are used in recurrent stenotic lesions after repeated PTA [6, 10]. The use of endovascular stents in hemodialysis vascular access continues to increase despite there being only 2 stents approved by the U.S. FDA to treat stenotic lesions; most of the uses seen in regular practice have been off label [10, 11, 12]. Some of the complications associated with stent placement include shortening, migration, and fracture [11] or permanent consequences in the ESKD life-plan and on future dialysis access creation [6, 7, 9, 11]. 

We present the case of a 40-year-old man with normal kidney function after kidney transplantation who had an episode of chest pain as a possible complication of a hemodialysis vascular access. 

## Case report 

### Clinical history and presentation of symptoms 

A 40-year-old male with medical history significant for hypertension, human immunodeficiency virus, and ESKD status post deceased donor kidney transplant who presented to the emergency department with a 1-week history of worsening sharp, non-radiating chest pain localized in the left hemithorax. Along with the pain, he was complaining of nausea and vomiting for 2 days for a total of 6 episodes. Emesis description was a thick mucus, possibly blood-streaked but without coffee-grounds. The patient stated he had never experienced this chest pain before. He reported not taking anything at home to reduce pain intensity. 

As part of his medical history, he was started on hemodialysis in 2015. He underwent the creation of a left brachial artery to brachial vein AVF in the mid-arm that did not mature, requiring a proximal brachial artery to brachial vein AVG using a 7-mm artery graft in a loop fashion. The patient underwent a deceased donor kidney transplant in January of 2020 and was off hemodialysis since. He had had multiple fistulograms with balloon angioplasty and a covered stent placement (fluency 10 mm) in January 2020 to keep the AVG patent. 

On physical examination, vital signs were normal, no skin lesions or tenderness to palpation on the chest wall, no cardiac murmurs, no lung abnormalities on auscultation, no renal graft tenderness without murmurs on auscultation, and patent AVG with good thrill and bruit, without physical findings of inflow or outflow stenosis. In the emergency room, he underwent laboratory analyses, resulting in two negative troponin levels and normal blood chemistry. 

### Imaging studies 

**Figure 1:**
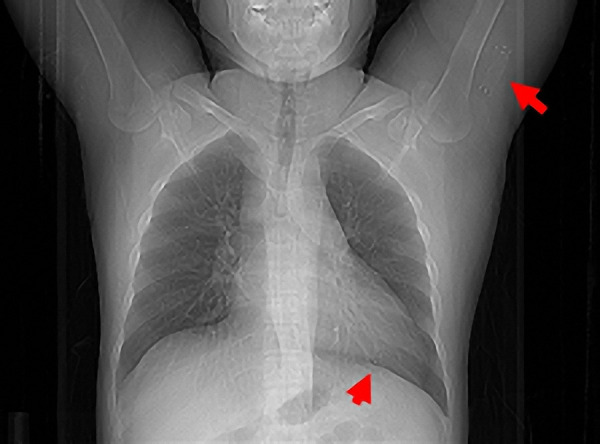
Chest X-ray demonstrating a venous stent in the left upper extremity with 4 heads in distal segment and 3 heads in proximal segment and a small metallic density over the left lung base (red arrows).

**Figure 2:**
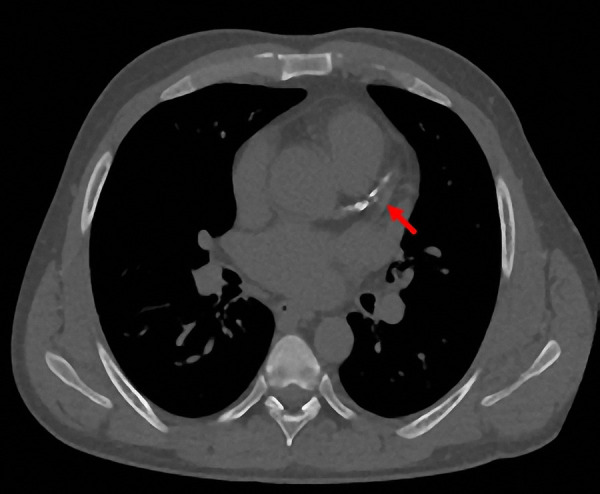
Computed tomography showing a small curvilinear metallic density at the right ventricular apex (red arrow).

**Figure 3:**
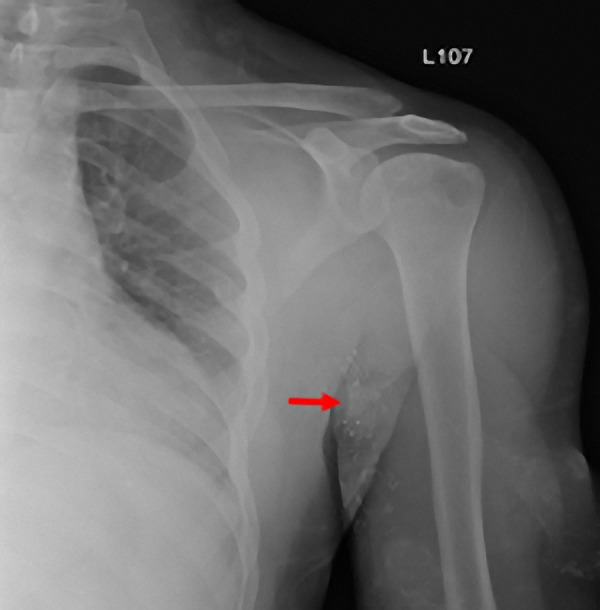
Incomplete venous stent in the left upper extremity as shown by X-ray (red arrow).

Serial electrocardiograms showed no ischemic changes. A two-dimension echocardiogram was negative for wall motion abnormalities, with preserved ejection fraction, and diastolic dysfunction grade II, with normal right ventricular systolic pressure. The chest X-ray reported a “curvilinear density projecting over the left lung base” (Figure 1), with a subsequent chest computed tomography (CT) reporting a “small curvilinear metallic density at the right ventricular apex with a total dimension of ~ 1 cm” (Figure 2). A left upper extremity X-ray showed a left arteriovenous access stent fracture, and all findings were consistent with an embolized fragment from the AVG stent (Figure 3). 

### Diagnosis 

The cause of the sent fracture was unclear and could have been caused by physical activity and/or a manufacturing issue. 

### Case resolution and follow-up 

The patient was not anticoagulated based on cardiology recommendations, as the stent fragment was similar to a pacemaker or defibrillator device, with no increased risk of embolization or clot formation. The stent was removed to avoid possible complications including new fragment migration or access complications. The patient underwent a left axillary vein stent explant with bovine patch revision of the left AVG. However, the embolized fragment could not be removed by CT-assisted surgery since it was a high-risk procedure. 

## Discussion 

In this report, we describe an uncommon case of non-cardiac chest pain secondary to a functional AVG complication. A multi-dimensional analysis is presented including AVG intervention history, imaging studies, and vascular access surgical revision. Arteriovenous grafts are normally the second vascular access choice for long-term hemodialysis when AVF are not an option or salvageable [13]. The primary failure rate for grafts is less than that for fistulas [14]. However, AVG frequently fail due to juxta-anastomotic stenosis that in most of the cases is believed to be related with neointimal hyperplasia, leading to thrombosis in 80% of the cases, requiring multiple interventions that contribute to the significant morbidity associated with dialysis-access complications [7]. A recent meta-analysis reported a primary patency and secondary patency at 2 years of 40% and 60%, respectively [15], but prior studies have shown a primary patency at 1 year from 22 to 42% for upper arm grafts AVGs [13]. These rates have been improving with endovascular treatments with stents, however, the main concerns regarding the use of stents are the limited areas of cannulation, stent fracture with or without migration, in-stent thrombosis, and limitation for future surgeries in the same extremity [8]. 

Stent fractures are commonly seen after deployment in arteries, but it is a rare event in the venous system [11]. One of the most commonly reported locations where dialysis endovascular stents can migrate is the costoclavicular junction; however, it can occur at any other location [16]. Finally, migration of an AVF or AVG stent to the heart is extremely uncommon [17], and is likely favored in this case by stent fracture. Nonetheless, this case demonstrates that hemodialysis vascular access complications like stent fracture and migration, with potential cardiovascular-related events, can be seen even when the access is no longer being actively cannulated. 

## Funding 

None. 

## Conflict of interest 

The authors declare that they have no relevant financial interests.
